# Infection Control in Hospitals of Jordan: Challenges and Opportunities

**DOI:** 10.7759/cureus.51328

**Published:** 2023-12-30

**Authors:** Mohammad Abu-Jeyyab, Batool Qura’an, Sallam Alrosan, Mohammad Al Mse'adeen

**Affiliations:** 1 School of Medicine, Mutah University, Al-Karak, JOR; 2 Surgery, Red Crescent Hospital, Amman, JOR; 3 Internal Medicine, Saint Luke’s Health System, Kansas City, USA

**Keywords:** antibiotic resistance, healthcare-associated infections, personal protective equipment (ppe), infection control policies, infection control measures, jordan

## Abstract

It is essential to take measures to prevent healthcare-associated infections (HAIs) and antibiotic resistance (AR) in order to ensure the safety of patients, control infections, protect public health, and maintain the overall quality and sustainability of the healthcare systems. The implementation of complex infection control strategies, the judicious utilization of antibiotics, health education, and global collaboration are necessary in order to address these significant challenges in the healthcare sector. In Jordan’s hospitals, infection control is a dynamic sector that is always adjusting to changing hazards and best practices due to the constant evolution of the profession. The nation's healthcare system strives to uphold high standards of hygiene and patient safety in order to achieve its goals of lowering the risk of infections that are linked with healthcare and protecting the general population's health. Hospitals in Jordan effectively manage infection control by using a multifaceted approach that includes regulation implementation, committees dedicated to the task, considerable training, and a variety of procedures. Patient, employee, and visitor safety are given first priority by these effective procedures. The careful application of personal protective equipment (PPE), strict isolation and quarantine procedures, well-defined visiting policies, pre-emptive vaccination campaigns, and thorough environmental cleaning procedures are essential elements of this strategy.

## Editorial

The current situation

Jordan has its own healthcare regulatory authority, the Health Care Accreditation Council (HCAC), which regulates hospital accreditation. Jordanian hospitals are supposed to meet HCAC standards for infection control and patient safety, which include hand hygiene, isolation precautions, personal protective equipment (PPE), environmental cleaning, sterilization, infection surveillance, vaccination, antibiotic stewardship, outbreak management, and patient and family education.

The World Health Organization (WHO) publishes global standards for infection prevention and control, which are frequently cited by Jordanian healthcare facilities [1]. In fact, in low- and middle-income countries such as Jordan, infection prevention and control policies are poorly adapted or insufficiently funded by governments. In Jordan, infection control committees or teams are typically in charge of monitoring and preventing healthcare-associated infections (HAIs). Infection control practitioners, nurses, and other healthcare workers are frequently included on these teams. HAI surveillance is a normal procedure, and data on infection rates are gathered, evaluated, and reported on a regular basis. To monitor infections, Jordanian hospitals are likely to have electronic health record (EHR) systems and reporting procedures in place. These systems aid in the rapid reporting and analysis of data. Jordanian hospitals utilize a variety of measures to assess infection control methods, including:

a. Healthcare-associated infection rates, such as surgical site infections. Surgical site infections affect 0.5% to 3% of surgical patients and are related to longer hospital stays than individuals who do not have surgical site infections [2].

b. Antibiotic resistance patterns and usage, including multidrug-resistant organism monitoring.

c. Healthcare personnel' adherence to hand hygiene practices.

d. Implementation of isolation precautions for contagious disease patients. Hospitals may also assess the proper usage of PPE as well as adherence to sterilization and disinfection regulations.

A case-control study was done in Jordan and confirmed the role of hand hygiene as one of the most cost-effective measures to combat the spreading of viral infections [3].

The challenges

In Jordan's hospitals, like many others worldwide, implementing and maintaining effective infection control faces numerous challenges. Each healthcare environment has unique problems; thus, infection control measures must be adapted to each setting. High-turnover outpatient settings may necessitate additional considerations, such as the establishment of patient triage and follow-up policies, as well as expanded cleaning and disinfection procedures [4]. These challenges include:

A. Resource constraints; hospitals frequently lack funds, staff, and suitable infrastructure, limiting access to vital infection control tools.

B. Inadequate training; healthcare staff may not obtain adequate infection control training.

C. High patient load; overcrowding makes maintaining spacing, isolation, and hygiene challenging.

D. In Jordan, some cultural factors might have an impact on infection control.

E. Limited surveillance; gaps in disease surveillance and uneven reporting make tracking and managing outbreaks difficult.

F. Treatment options are limited: As bacteria, viruses, and other infections develop resistance to routinely used antibiotics and antimicrobial medicines, Jordan's healthcare providers may have less effective treatment alternatives. This can make infection treatment more challenging, resulting in longer hospital stays, higher healthcare expenses, and a higher likelihood of treatment failure.

G. Inadequate infrastructure; in older hospitals, ventilation, hygiene stations, and sanitation facilities may be lacking.

The opportunities

Implementation of prevention bundles, increased government commitment, improved hand hygiene compliance, surveillance, prudent antimicrobial use, translation of research results into practice, and upgrading the capabilities of microbiology laboratories are what are considered to be the most effective solutions for an infection control program that is effective. Putting more of an emphasis on infection control is one way that nations with limited resources might enhance the quality of healthcare in the future [5].

It is possible to improve infection control in Jordan by engaging in certain activities, such as increasing awareness and education among healthcare professionals, patients, and the general public on the need of infection control.

1. The development of novel approaches to infection control can be facilitated by fostering research and fostering partnerships with educational institutions and research institutes.

2. In addition to increasing the effectiveness of hospital leadership and governance, this can also play a significant role.

Recommendations

Recommendations and suggestions for Jordanian hospital administrators, health professionals, and policymakers should be based on a combination of the best available evidence and the local circumstances. Some of these recommendations are:

I. Hand hygiene programs

II. Isolation precautions

III. Lab infection control

IV. Respiratory hygiene and cough etiquette

V. Surgical site infection prevention

VI. Collaboration with public health authorities

## References

[1] Balakrishnan VS (2022). WHO's first global infection prevention and control report. Lancet Infect Dis.

[2] Seidelman JL, Mantyh CR, Anderson DJ (2023). Surgical site infection prevention: a review. JAMA.

[3] Tarif AB, Ramadan M, Yin M (2022). Infection prevention and control risk factors in health workers infected with SARS-CoV-2 in Jordan: a case control study. PLoS One.

[4] Sturm L, Flood M, Montoya A, Mody L, Cassone M (2021). Updates on infection control in alternative health care settings. Infect Dis Clin North Am.

[5] Raka L (2010). Prevention and control of hospital-related infections in low and middle income countries. Open Infect Dis J.

